# Exploring Molecular Targets of Quercetin for the Treatment of Nicotine‐Related Oral Carcinoma: A Network Pharmacology Analysis and In Vitro Study

**DOI:** 10.1002/fsn3.71241

**Published:** 2025-12-08

**Authors:** Xiaopeng Guo, Zhen Sun, Huarong Chen, Changya Li, Aoshuang Chang, Houyu Zhao, Junjun Ling, Xianlu Zhuo

**Affiliations:** ^1^ Affiliated Hospital of Guizhou Medical University Guiyang Guizhou China

**Keywords:** network pharmacology, nicotine, oral carcinoma, quercetin

## Abstract

Nicotine, which is enriched in tobacco, has been identified as an important factor in the development of oral cancer. The natural flavonoid quercetin has potential anti‐tumor properties due to its low toxicity and high efficacy. We aimed to explore the potential molecular targets of quercetin for the treatment of nicotine‐related oral cancer by network pharmacological analysis and to evaluate its efficacy in vitro experiments. A total of 29 potential target genes were identified, which may be associated with epithelial–mesenchymal transition (EMT), the receptor tyrosine kinase (RTK) pathway, and immune cell infiltration, as well as acquired resistance to various chemotherapeutic agents. Molecular docking and molecular dynamics simulation indicated that quercetin may bind more strongly to potential key genes (THBS1, SERPINE1, and IGF1R). Quercetin was shown to affect key gene expressions in nicotine‐related oral cancer cells and attenuate their malignant phenotype in vitro experiments. A series of novel targets for quercetin in the treatment of nicotine‐related oral cancer were identified. These findings not only help understand the pathogenesis of oral cancer but also help explore its precancerous preventive measures, which are of great value for oral cancer prevention.

## Graphical abstract

Molecular docking and molecular dynamics simulations revealed that quercetin binds tightly to the core targets (THBS1, SERPINE1, and IGF1R) of nicotine‐related oral carcinoma, suggesting its potential as a therapeutic agent for oral cancer. In vitro experiments demonstrated that quercetin can inhibit the expression of the core targets (THBS1, SERPINE1, and IGF1R), thereby suppressing the proliferation and metastasis of both nicotine‐transformed cells and oral cancer cells.

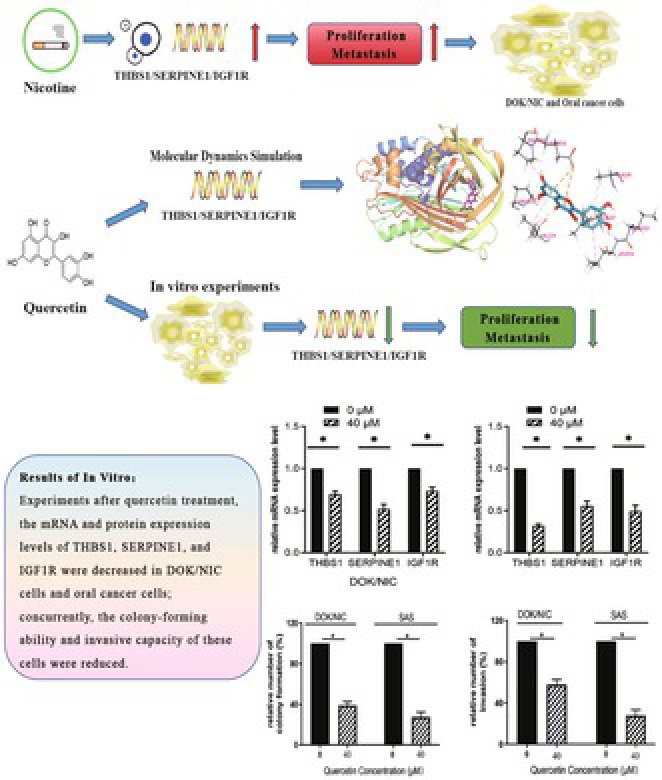

## 1 Introduction

Oral cancer, which is a prevalent form of head and neck cancer (HNC), is among the top ten most prevalent malignant tumors globally. It primarily occurs in the oral cavity and adjacent tissues, and its incidence has been steadily increasing (Lima et al. 2021). Distressingly, a considerable number of oral cancer patients are diagnosed at an advanced stage, and despite undergoing surgery, radiotherapy, and chemotherapy, their 5‐year survival rate remains low, at less than 50% (Dholariya et al. 2023; Goel et al. 2022). Consequently, there is an urgent clinical need to explore alternative approaches for the prevention and treatment of oral cancer.

Scientific evidence has shown that smoking is the primary causative factor of several illnesses, including oral cancer, which accounts for more than 8 million deaths worldwide (Ahmed et al. 2021). Nicotine, a principal active compound found in tobacco, is believed to contribute significantly to the carcinogenesis of oral cancer (Chien et al. 2021). Research has demonstrated that the binding of nicotine to nAChR promotes various malignant phenotypes, such as proliferation, migration, and invasion of oral cancer cells (Sharma et al. 2022). Our prior investigation has established nicotine‐induced malignant oral epithelial cells and identified SERPINE1 as being a crucial gene involved in the malignant transformation processes (Guo et al. 2023). Thus, chronic exposure to nicotine results in the malignant transformation of oral epithelial cells, which may eventually lead to oral cancer. If the malignant process of the cells can be inhibited, it is expected that the incidence of oral cancer due to nicotine exposure can be greatly reduced. However, there are currently no clinically effective drugs that can intervene in this process. As the carcinogenesis of nicotine exposure is a long process, it is particularly important to find less toxic and more effective drugs to intervene in this process for cancer prevention and treatment.

Natural products have distinct advantages in tumor treatment, including their low toxicity, reduced side effects, and potent anti‐tumor activity (Talib et al. 2022). These substances can help mitigate the adverse effects and complications associated with chemotherapy, radiotherapy, and targeted therapies, and can be chosen to alleviate symptoms and enhance the quality of life for cancer patients (Dasari et al. 2022). For instance, Cortex Mori induces apoptosis and inhibits tumor invasion in melanoma cells by blocking PI3K/AKT signaling (Hu et al. 2022).

Quercetin, a flavonol compound derived from natural plants, is primarily found in vegetables and fruits such as onions, apples, and cherries. It exhibits a range of activities, including antiviral, anti‐inflammatory, antioxidant, and anti‐tumor effects (Di Petrillo et al. 2022). In particular, quercetin has been shown to effectively inhibit varicella‐zoster virus and HCMV replication (Kim et al. 2020), enhance antioxidant capacity and glycogen storage and improve muscle function to promote anti‐fatigue ability (Chen et al. 2021). Recently, its potential as an anticancer agent has gained significant attention. Specifically, quercetin has been reported to inhibit tumor cell proliferation and invasion (Reyes‐Farias and Carrasco‐Pozo 2019), possibly by regulating multiple signaling pathways such as Akt‐mTOR (Jia et al. 2018), PI3K/AKT (Maurya and Vinayak 2015), SIRT1/AMPK (Guo et al. 2021), MAPK, and WNT (Kedhari Sundaram et al. 2019). A study even suggested its inhibitory effects on oral cancer cells (Son and Kim 2023). In the preliminary experiment, we were surprised to find that quercetin inhibited, among other things, the proliferation of nicotine‐transformed oral epithelial cells. However, the mechanism by which quercetin intervenes in the carcinogenic process of nicotine is poorly understood. Elucidating the molecular mechanism is important for understanding the biological activity of quercetin and exploring its clinical application.

Traditional studies often focus on a single pathway or target, neglecting the intricate and interconnected nature of biological processes. However, network pharmacology, which combines systems biology with pharmacology and biological networks, has emerged as a powerful approach that replaces the traditional one‐target‐one‐drug model with a network‐based, multi‐component, and multi‐pathway model (Luo et al. 2020). For instance, a network pharmacology analysis has been used to screen numerous targets and pathways in the treatment of non‐small cell lung cancer with Fisetin (Ling, Wang, et al. 2022).

This study aimed to investigate the potential anticancer gene targets of quercetin in nicotine‐associated oral cancers from multiple levels and perspectives using the in silico approach. In addition, the interventional effects of quercetin on nicotine‐induced malignantly transformed cells in the oral epithelium were further investigated by in vitro experiments. Overall, this study is expected to provide new insights into quercetin's role in inhibiting the malignant transformation of oral epithelial cells and offer evidence for the primary prevention of oral cancer.

## 2 Materials and Methods

### 2.1 Analysis of Pharmacokinetic Parameters of Quercetin

Traditional Chinese Medicine Systems Pharmacology (TCMSP) Database (https://old.tcmsp‐e.com/tcmsp.php) is a publicly accessible platform for analyzing Chinese herbal medicines, containing data related to herbs‐composition‐disease‐target. This database also provides detailed information on 12 important ADME‐related properties, including oral bioavailability, half‐life, drug similarity, permeability, and blood‐brain barrier penetration, as well as drug targets and diseases associated with each active compound (Ru et al. 2014). Furthermore, the TCMSP database provides detailed information on the drug targets and diseases that are associated with each of the active ingredients, including quercetin. As a result, we were able to obtain valuable pharmacokinetic parameters for quercetin from this database.

### 2.2 Screening Targets for Quercetin in Treating Nicotine‐Related Oral Carcinoma

#### 2.2.1 Screening for the Target Genes of Quercetin

Target genes of quercetin were obtained through three methods. Firstly, the target genes/proteins of quercetin were directly retrieved from the TCMSP platform. The protein names were then converted into corresponding gene names using the UniProt database (UniProt: The Universal Protein Knowledgebase in 2021 2021). Secondly, target gene prediction of quercetin was performed using the CTD (Davis et al. 2021) and Herb databases (Fang et al. 2021), respectively. The selection cutoff points in the CTD database was set as an interaction count greater than 1. Finally, all potential target genes were collected and duplicate entries were removed to create a unique gene set.

#### 2.2.2 Screening for the Hub Genes of Nicotine‐Related Oral Carcinoma

The screening process was thoroughly described in our recent study (Guo et al. 2023). To identify hub genes associated with nicotine‐related oral carcinoma, we analyzed the publicly available dataset GSE89923 (Woo et al. 2017).

#### 2.2.3 Screening for Common Gene Targets

To identify the common target genes between nicotine‐related oral carcinoma and quercetin, a Venn analysis was employed. The gene targets that were identified in the intersection of these two sets can be considered potential targets for quercetin in the treatment of nicotine‐related oral carcinoma.

### 2.3 Function Annotation of the Common Gene Set in HNC

#### 2.3.1 Gene Ontology (GO) and Kyoto Encyclopedia of Genes and Genomes (KEGG) Enrichment Analysis of the Gene Set

To gain insight into the biological functions of the target genes, the GO analysis (Ashburner et al. 2000) was conducted. In addition, the KEGG analysis (Kanehisa and Goto 2000) was used to identify potential enrichment signaling pathways associated with these genes. *p* < 0.05 was considered statistically significant.

#### 2.3.2 Assessment of the Relationship Between the Common Gene Set and the Malignant Phenotype of HNC

To explore the potential functions of these crossover genes, the GSCA platform (Liu et al. 2023) was utilized for analysis. This platform is a TCGA database‐based analysis tool that integrates data from 33 different cancer types, including gene expression, mutations, drug sensitivity, and more. One of the main advantages of this platform, in comparison to other platforms that only involve single gene analysis, is its ability to perform genomic analysis. This feature enables a comprehensive understanding of the collective function of multiple genes toward elucidating the potential biological role of specific genomes. Our analysis primarily focused on HNC‐related data within this platform, evaluating the interplay between gene set expression in HNC, immune cell infiltration, drug sensitivity, and other relevant factors.

### 2.4 Construction of the Interplay Network and Molecular Docking

#### 2.4.1 Establishment of the Diseases‐Targets‐Functional Annotations‐Signaling Pathways Network

The network interaction model, which includes diseases, targets, functional annotations, and signaling pathways, was developed to illustrate the potential connections among quercetin, nicotine‐related oral carcinoma, biological processes, and target genes.

#### 2.4.2 Construction of Protein–Protein Interaction (PPI) Networks

There is a possibility of numerous interactions among genes/proteins, and an effective approach to comprehending these relationships is through the use of PPI networks. The STRING database (Szklarczyk et al. 2021) was utilized to predict and analyze protein interactions, as it is a user‐friendly web server capable of constructing composite gene–gene function interaction networks from gene lists. Cytohubba is a Cytoscape plugin that calculates the degree and betweenness values of each node within a PPI network (Chin et al. 2014). The higher the score, the more crucial a role the node may have in the network. The Cytohubba tool is applied to visualize PPI networks.

#### 2.4.3 Molecular Docking

The genes that were ranked as the top candidates in the PPI network and that showed a correlation with prognosis were selected to carry out molecular docking with quercetin. The molecular docking was performed using the PyMOL and Autodock tool (Seeliger and de Groot 2010). The 2D structure of quercetin was retrieved from the PubChem website (Kim et al. 2021) and converted to the Mol2 format using the Chem3D software. As for the candidate protein, its crystal structure was obtained from the RCSB PDB database before docking, ligand molecules and water molecules were removed from the protein structure using the PyMol software. Finally, the docking results were imported into the PyMol software for further analysis and visualization.

#### 2.4.4 Molecular Dynamics (MD) Simulation

Based on the molecular docking results, molecular dynamics simulations were performed using the Gromacs2022 software package. The small molecule ligand was described by the GAFF force field, while the protein was treated with the AMBER14SB force field, along with the TIP3P water model. The protein and ligand coordinate files were merged to construct the simulation system for the complex. Simulations were conducted under isothermal–isobaric conditions with periodic boundary constraints. Throughout the MD simulations, all bonds involving hydrogen atoms were constrained using the LINCS algorithm, with an integration time step of 2 fs. Electrostatic interactions were calculated using the Particle Mesh Ewald (PME) method with a cutoff of 1.2 nm. The non‐bonded interaction cutoff was set to 10 Å and updated every 10 steps. The V‐rescale thermostat was employed to maintain the temperature at 298 K, and the Berendsen barostat was used to regulate the pressure at 1 bar. The system underwent 100 ps of equilibration under NVT and NPT ensembles at 298 K, followed by a 100 ns production MD simulation. Conformations were saved every 10 ps. After simulation, the trajectories were analyzed using VMD and PyMOL. The binding free energy between the protein and the ligand was calculated using the g_mmpbsa tool based on the MM‐PBSA approach.

### 2.5 Validation of Hub Targets Through in Vitro Experiments

#### 2.5.1 Cell Culture

Nicotine‐induced transformed oral epithelial cells, referred to as DOK/NIC, were effectively established in a previous study (X. Guo et al. 2023) and have been stored in our laboratory for further research. Both DOK/NIC cells and the SAS oral cancer cell line were cultured in Dulbecco's modified Eagle's medium supplemented with 10% fetal bovine serum and maintained at a temperature of 37°C in a humidified atmosphere with 5% CO_2_.

#### 2.5.2 Expression of Key Genes in DOK/NIC and SAS Oral Cancer Cells

The mRNA and protein expression levels of key genes in the post‐intervention cells were assessed using qRT‐PCR and Western blot, respectively, according to the previously described protocols (Ling, Zhang, et al. 2022). Each sample was analyzed in triplicate, and GAPDH was utilized as an internal reference.

Following are primers used in this study:

THBS1: (F: AGACTCCGCATCGCAAAGG; R: TCACCACGTTGTTGTCAAGGG).

SERPINE1: (F: ACCGCAACGTGGTTTTCTCA; R: TTGAATCCCATAGCTGCTTGAAT).

IGF1R: (F: TCGACATCCGCAACGACTATC; R: CCAGGGCGTAGTTGTAGAAGAG).

GAPDH: (F: AAGGTCGGAGTCAACGGATTTG; R: CATGGGTGGAATCATATTGGAA).

#### 2.5.3 CCK8 Assay and Colony Formation Assay

Cell viability was determined by Cell Counting Kit‐8 (CCK‐8) (Immunopath Biotechnology, USA). Cells were seeded in 96‐well culture plates at a density of 4 × 10^3^ cells per well during the logarithmic growth phase. After treatment with different concentrations of quercetin for 24 and 48 h, the CCK‐8 reagent was added to the plates, and the optical density (OD) values at 450 nm were measured. Colony formation assays were then performed to assess the clonogenic capacity of the cells. The cells were seeded in 6‐well plates at a density of 600 cells per well and incubated at 37°C in a humidified atmosphere containing 5% CO2 for 10 days. Clonogenic potential was determined by staining the cells with crystal violet and counting the number of colonies formed.

#### 2.5.4 Transwell Invasion Assay

A cell suspension of 4 × 10^4^ cells/mL was prepared in serum‐free medium after the indicated treatment. The cell suspension was then added to the upper chamber of 24‐well Transwell inserts (8 μm pore size, corning, USA) covered with matrix gel (BD, USA). Subsequently, the lower chamber was filled with 600 μL RPMI 1640 supplemented with 20% FBS. The apparatus was then incubated at 37°C for 18 h to allow for the invasion assay. Following the incubation period, the cells adhered inside to the upper chamber were removed, and the cells on the outer side of the bottom filter were stained with Jimsa (Sigma‐Aldrich, St. Louis, MO) for 10 min. Subsequently, images were captured, and the cell count was determined.

### 2.6 Statistical Analysis

For continuous variables, we employed *t*‐tests, ANOVA, or Wilcoxon rank‐sum tests, depending on the nature of the data. When evaluating ratio comparisons, the chi‐square test was selected. Survival analysis was conducted using the Kaplan–Meier method to generate overall survival curves, while the log‐rank test was employed to establish discrepancies in survival rates. These analyses were performed using MedCalc software. A significance level of *p* < 0.05 was used to determine statistical significance.

## 3 Result

**FIGURE 1:**
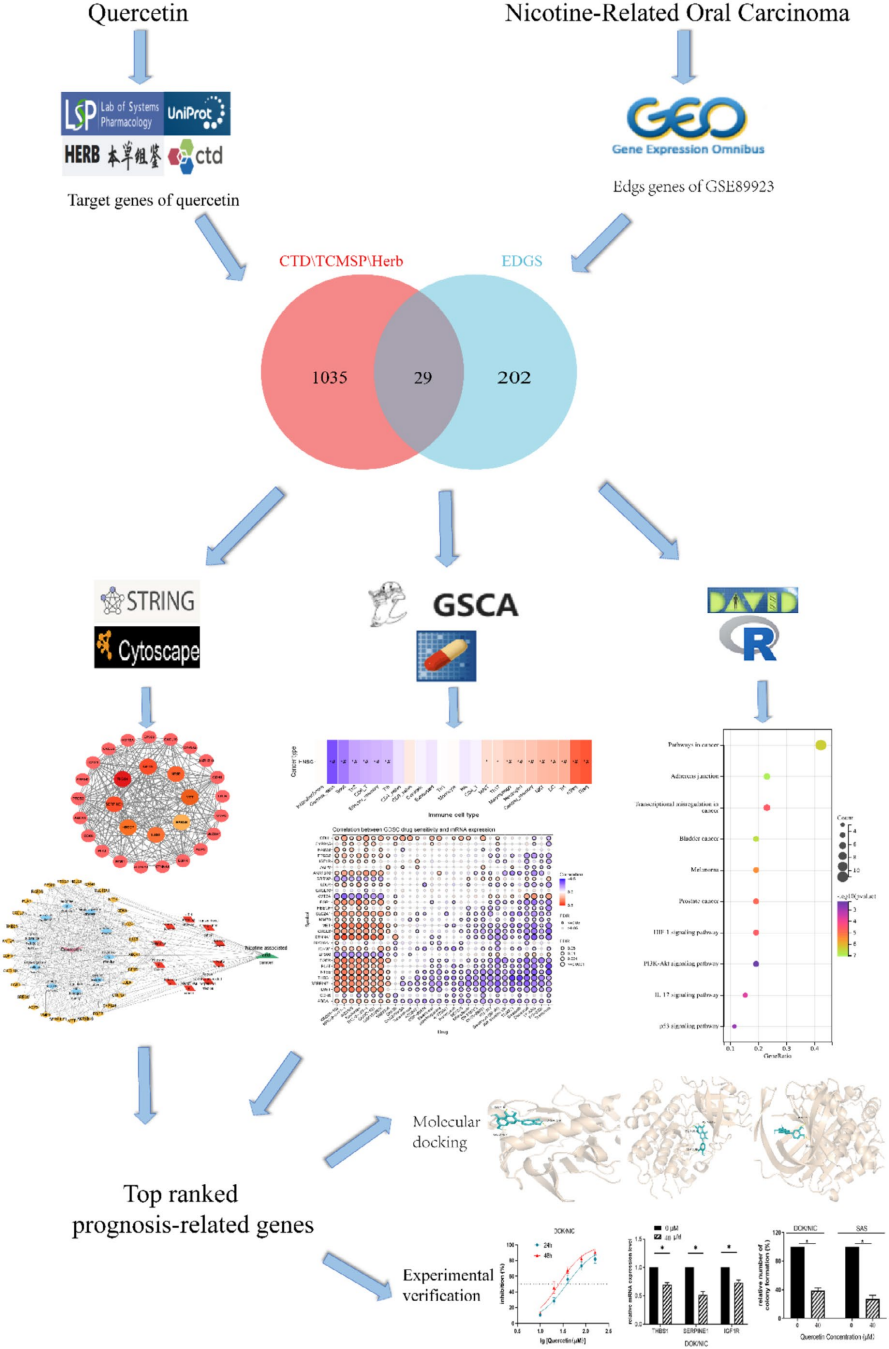
Research roadmap for this study. A research flowchart on the intervention of quercetin in nicotine‐related oral cancer, with each graph representing some tools and results used in the study.

Quercetin is a flavonoid compound found in herbs, vegetables, and fruits. Its potential anticancer activity has been widely studied. This research investigates the use of quercetin in treating nicotine‐related oral carcinoma (Figure 1). A total of 29 genes shared by quercetin and nicotine‐related oral carcinoma were screened, and SERPINE1, IGF1R, and THBS1 were identified as hub genes. Further molecular docking and in vitro analysis confirmed these results.

### 3.1 Pharmacokinetic Parameters of Quercetin

**FIGURE 2:**
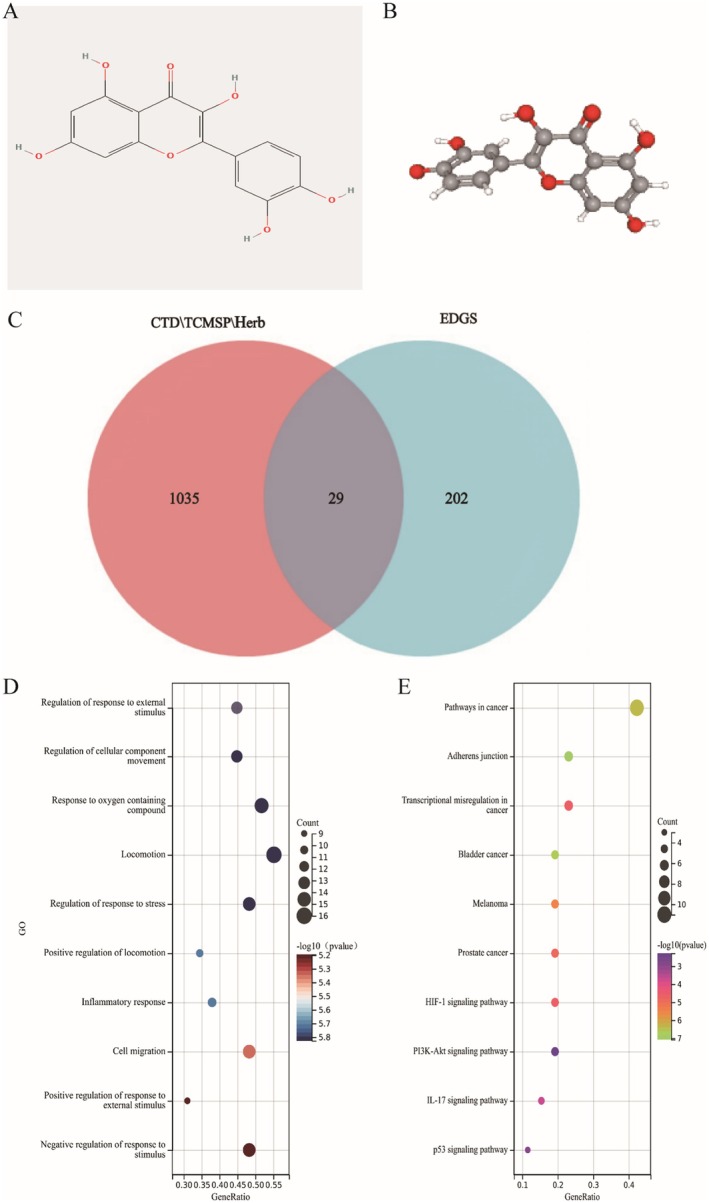
The chemical structure of quercetin and enrichment analysis of the common target genes. (A, B) The 2D and 3D structure of quercetin. (C) Twenty‐nine genes shared by quercetin and nicotine‐related oral carcinoma were obtained by Venn analysis. (D) The top GO enrichment analysis terms of the common target genes. (E) The top KEGG enrichment analysis terms of the common target genes.

The pharmacokinetic parameters of quercetin were obtained from the TCMSP database. The 2D and 3D structures of quercetin are presented in Figure 2A,B respectively. Quercetin has a molecular ID of MOL0000989, a molecular formula of C15H10O7, a relative molecular weight of 302.25, a lipid‐water partition coefficient (AlogP) of 1.50, an oral bioavailability (OB) of 46.43, a blood–brain barrier (BBB) of −0.77, and a drug similarity (DL) of 0.28. It is worth noting that OB ≥ 30 and DL ≥ 0.18 are commonly used as screening criteria for bioactive ingredients (Liu et al. 2013). Quercetin successfully met these screening criteria.

### 3.2 Screening of Common Targets and Assessment of Their Role in HNC

#### 3.2.1 Screening of the Common Targets of Quercetin and Nicotine‐Related Oral Carcinoma

Firstly, the targets of quercetin were screened. One hundred and fifty‐four possible targets were obtained from the TCMSP database, 161 targets from the Herb database, and 998 potential targets by setting the Interaction Count greater than one as the selection criterion from the CTD database. A collection of 1064 target genes was acquired. Secondly, an analysis of the GSE89923 dataset led to the identification of 232 genes associated with nicotine‐related oral carcinoma. Thirdly, a Venn analysis was performed, revealing 29 genes that were common between quercetin and nicotine‐related oral carcinoma (Figure 2C). These 29 genes are as follows: RAB3B, KMT2A, SRRM2, MET, CTNNA1, IL6ST, NT5E, HBEGF, PLAT, CDH1, AQP3, AKR1B10, LDLR, SLC2A1, SLC16A1, PTGS2, CXCL8, CXCL10, MMP3, EGFR, IGF1R, CDK6, ICAM1, EP300, THBS1, EGR1, SERPINE1, CYP3A5, and ABCA1.

#### 3.2.2 Functional Enrichment of the Common Genes

We performed GO and KEGG pathway enrichment analyses on the shared genes, with the former encompassing Biological Process (BP), Cellular Component (CC), and Molecular Function (MF). In this study, we focused our analysis on the most frequently implicated biological process. Based on a *p* < 0.05, we found that 875 GO terms were associated with these genes. Figure 2D presents the top 10 GO terms. KEGG pathway enrichment analysis showed that these genes were enriched in 58 pathways, with Figure 2E listing the highest‐ranking signaling pathway terms such as Pathways in cancer, Transcriptional misregulation, and PI3K‐AKT signaling pathway. These findings suggest that these pathways may be involved in the intervention of quercetin in oral cancer.

#### 3.2.3 Expression and Prognostic Relevance of the Common Genes in HNC

**FIGURE 3:**
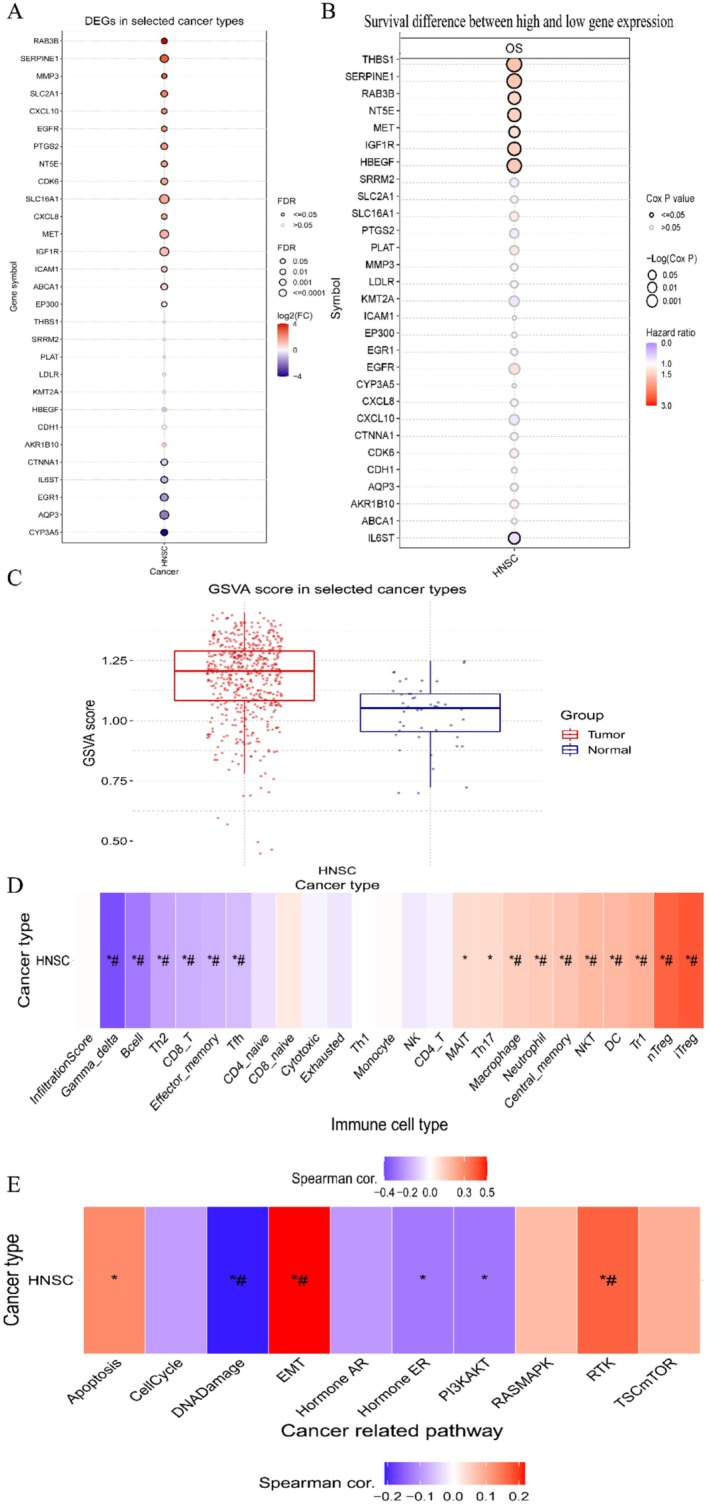
Analyzing the common target genes based on the GDSC database. (A) Analysis of the expression of the common genes in HNC based on the GDSC database. Orange represents high expression in tumor tissues, and basket color represents low expression. (B) Relationship between common genes and prognosis of HNC patients. Orange represents negative correlation with prognosis. Vice versa is positive correlation, and solid circle represents statistically significant (*p* < 0.05). (C) GSVA scores of HNC tumor tissues and normal tissues were performed separately, and the GSVA score of tumor tissues was higher than that of normal tissues (*p* < 0.05). (D) The relationship between the common genes and immune cell infiltration was analyzed based on the GSVA scores. (E) The association of the common genes with certain pathways was analyzed based on the GSVA scores, in which orange represents positive association, purple represents negative association, and the darker color represents the greater association. *p < 0.05; #: FDR < 0.05.

To investigate the expression and prognostic relevance of the common genes in head and neck cancer (HNC), we assessed the HNC cohort in the TCGA database. As depicted in Figure 3A, RAB3B, SERPINE1, MMP3, SLC2A1, CXCL10, EGFR, PTGS2, NT5E, CDK6, SLC16A1, CXCL8, MET, IGF1R, ICAM1, ABCA1, and EP300 were identified as highly expressed in HNC tumors (*p* < 0.05), while CTNNA1, IL6ST, EGR1, AQP3, and CYP3A5 were determined to be downregulated (*p* < 0.05). The expression of the remaining genes did not exhibit any significant difference. Specifically, THBS1, SERPINE1, RAB3B, NT5E, MET, IGF1R, HBEGF, and IL6ST were identified as being associated with prognosis (*p* < 0.05, Figure 3B).

Furthermore, we conducted Gene Set Variation Analysis (GSVA) using the aforementioned 29 common genes as a gene set. The GSVA score represents the comprehensive level of gene set expression and positively correlates with the expression of the gene set. As depicted in Figure 3C, the GSVA scores of hub genes in tumor tissues were significantly higher compared to those in normal tissues.

#### 3.2.4 Relationship Between the Common Genes and Immune Infiltration

To investigate the relationship between common genes and immune cell infiltration, we conducted a comprehensive assessment of the association between the overall expression of the common genes (GSVA scores) and 24 immune cell types. As shown in Figure 3D, the expression of hub genes was positively correlated with MAIT, Th17, Macrophage, Neutrophil, Central‐memory, NKT, DC, Tr1, nTreg, and iTreg cells. On the other hand, it was negatively correlated with B cells, CD8‐T cells, Gamma‐delta cells, Th2 cells, Effector‐memory cells, and Tfh cells.

#### 3.2.5 The Relationship Between the Common Genes and Pathways

**FIGURE 4:**
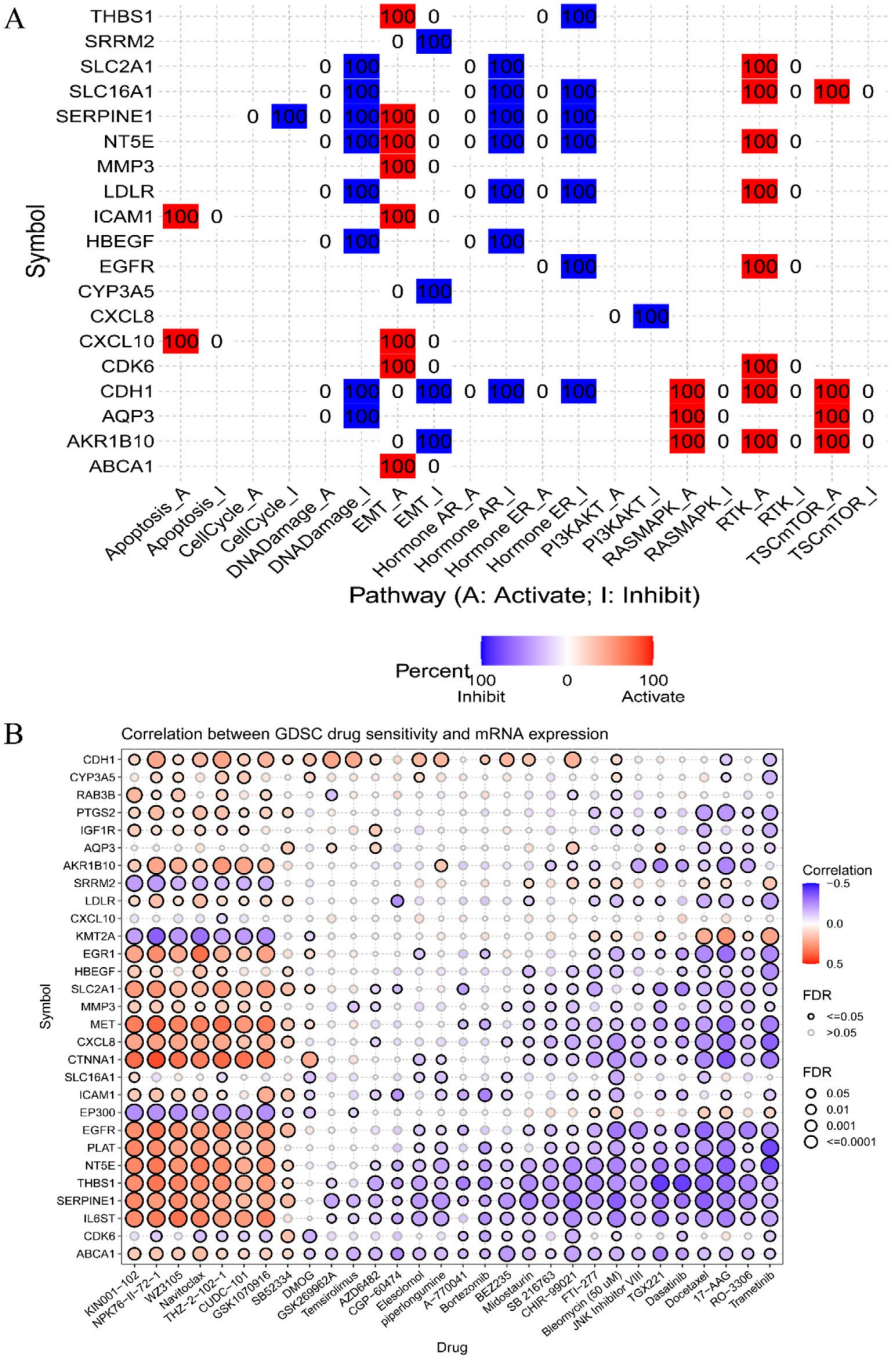
The results of the relationship between the common target genes and pathways and drug sensitivity. (A) Correlation of 29 common genes with some pathways, where red represents positive correlation, and blue represents negative correlation. (B) Correlation of 29 genes with chemotherapeutic drug sensitivities based on the GDSC database, where red represents positive correlation, blue‐purple represents negative correlation, darker color represents stronger correlation, and solid line represents FDR < 0.05.

To gain a deeper understanding of the function of common genes, we analyzed the relationship between common genes and some pathways and functions. As shown in Figure 4A, certain genes are positively correlated with EMT, RTK, TCS‐mTOR, and RAS‐MAPK, while they are negatively correlated with DNA Damage, EMT, Hormone AR, and Hormone ER. Moreover, we performed a GSVA analysis, and the results are illustrated in Figure 3E. It was found that the GSVA scores are positively correlated with EMT, and RTK pathways, and negatively correlated with DNA Damage, which is consistent with the results of the analysis of individual genes.

#### 3.2.6 The Relationship Between the Common Genes and Drug Sensitivity

To predict the influence of the common gene expression on the sensitivity of cancer cells to chemotherapy, we evaluated the data from the GDSC database. As shown in Figure 4B, it revealed a negative correlation between the expression of most genes and sensitivity to multiple drugs, including Dasatinib, RO‐3306, Trametinib, 17‐AAG, Bleomycin, and Docetaxel. Among them, Bleomycin and Docetaxel are used clinically for oral cancer treatment. From this, we speculate that quercetin may improve the sensitivity of cancer cells to chemotherapy drugs by regulating these common genes.

### 3.3 Establishment of the Interplay Network and Molecular Docking

#### 3.3.1 Construction of the Diseases‐Targets‐Functional Annotations‐Signaling Pathways Network

**FIGURE 5:**
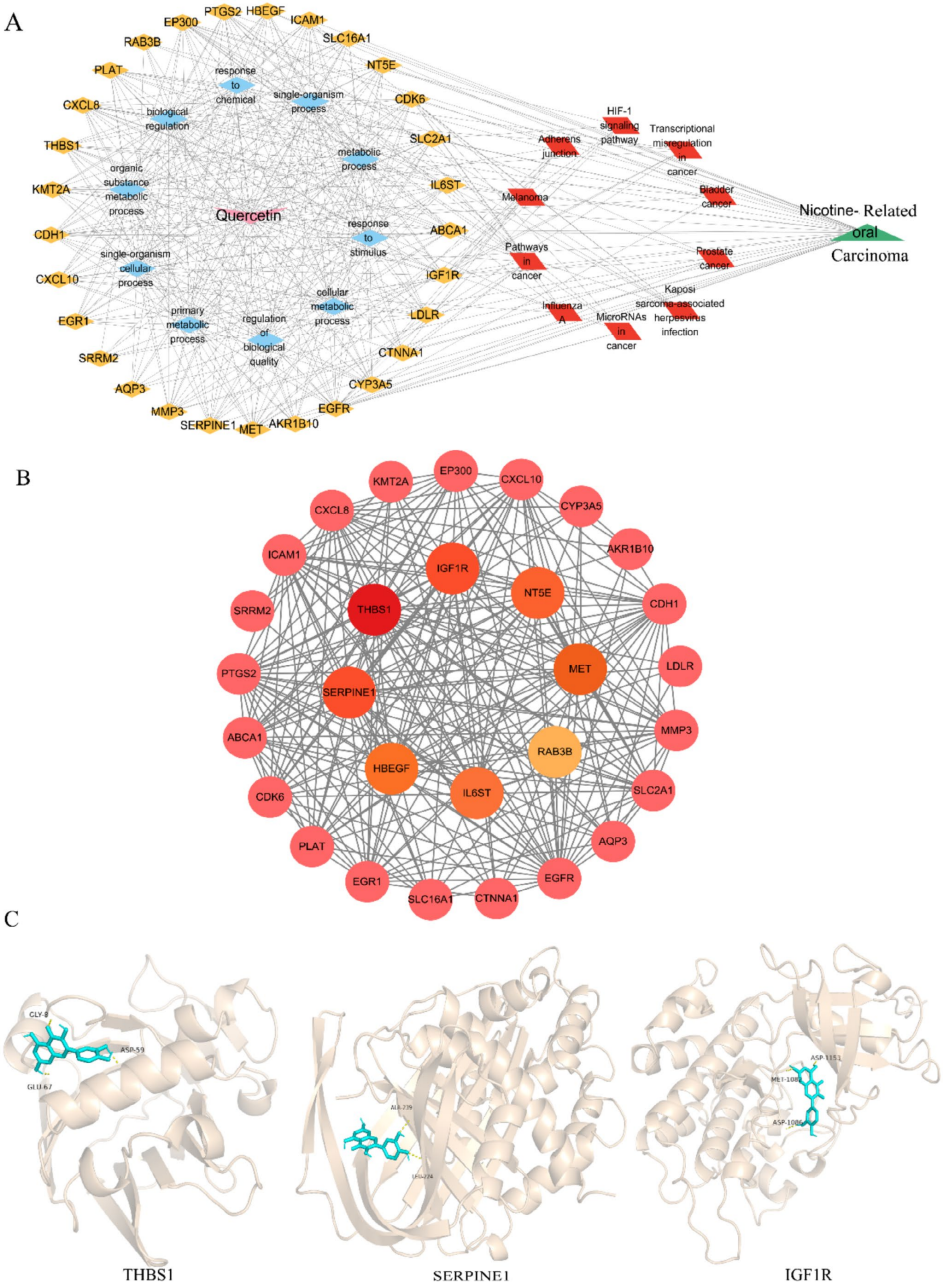
The interplay network and molecular docking. (A) The Diseases‐target functional annotations‐signaling pathways network. (B) The PPI network of the common target genes and their interactions. (C) The molecular docking results of quercetin with THBS1, SERPINE1, and IGF1R are graphically visualized, with the green structure representing quercetin and the yellow dashed line representing the connecting bond between quercetin and protein. The gray ribbon represents the structure of the gene protein.

A network model was constructed for visualization. Figure 5A shows the network containing quercetin, disease (nicotine‐related oral carcinoma), biological process ‐signaling pathways, and the target genes, which highlights the relationships among these entities. The lines represent the interaction between the two.

#### 3.3.2 Protein–Protein Interaction (PPI) Networks

During tumor development, multiple genes/proteins interact with one another to construct intricate regulatory networks, enabling them to collaborate in executing diverse biological processes. The PPI network was visualized using Cytoscape. The PPI network illustrated in Figure 5B comprises 29 nodes, with each node representing a gene/protein, and each edge denoting an interaction among proteins. Table 1 ranks the genes related to HNC prognosis according to their degree. The top three genes (THBS1, SERPINE1, and IGF1R) were selected for molecular docking and experimental validation.

**TABLE 1:** Genes associated with prognosis sorted by degree.

Node‐name	Degree	Expression in HNC	*p*
THBS1	44	High	0.0004
SERPINE1	42	High	0.0009
IGF1R	40	High	0.0048
MET	38	High	0.0392
HBEGF	34	High	0.0012
NT5E	32	High	0.0049
IL6ST	30	Low	0.0213
RAB3B	2	High	0.0105

#### 3.3.3 Molecular Docking

Docking was performed between the candidate core proteins (THBS1, SERPINE1, and IGF1R) and quercetin. Molecules and ligands could effectively bind to each other if the binding energy was less than zero, which was generally considered less than −5. Table 2 presents the binding affinities between these proteins and quercetin, ranging from −7.6 to −7.2 Kcal/mol. As shown in Figure 5C quercetin has an excellent binding ability to the candidate proteins.

**TABLE 2:** The molecular docking results.

Node_name	Protein	PDB identifier	Estimated ΔG (kcal/mol)
THBS1	Thrombospondin‐1	2ERF	−7.2
SERPINE1	SERPINE1 mRNA‐binding protein 1	1LJ5	−7.6
IGF1R	Insulin‐like growth factor 1 receptor	5FXS	−7.6

#### 3.3.4 Molecular Dynamics Simulation

**FIGURE 6:**
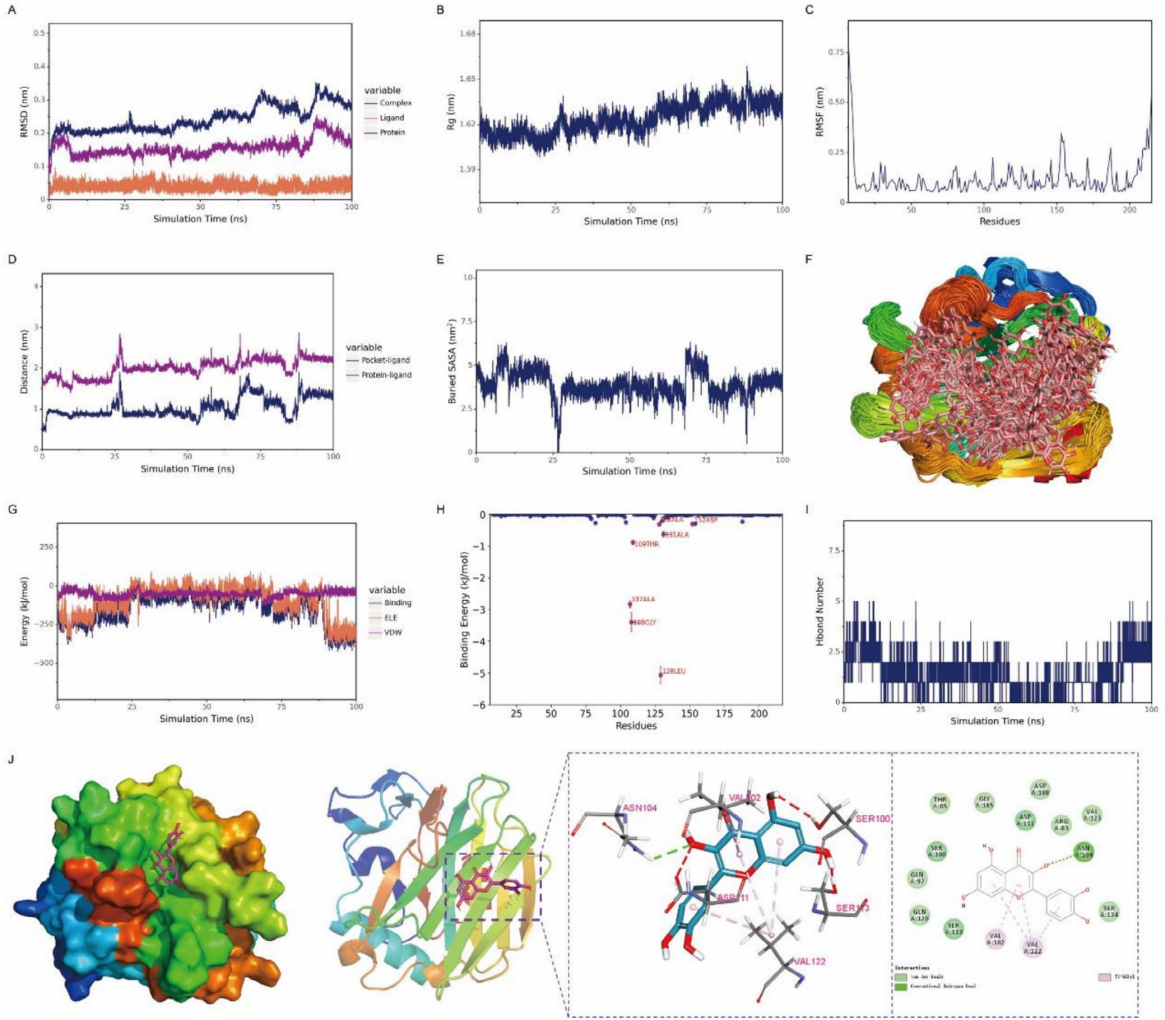
Molecular dynamics simulations demonstrate the stable binding of quercetin to the THBS1 protein. (A) Root‐mean‐square deviation (RMSD) of the complex, protein, and ligand; (B) Radius of gyration (Rg) of the complex; (C) Root‐mean‐square fluctuation (RMSF) of protein residues; (D) Protein‐ligand binding site distance; (E) Buried solvent‐accessible surface area (SASA); (F) Superimposed conformational snapshots; (G) van der Waals (VDW) and electrostatic (ELE) energy components; (H) Binding energy contribution per residue; (I) Hydrogen bond number; (J) Molecular interaction diagram.

Quercetin formed a stable binding with the THBS1 protein, and the complex structure became progressively stabilized over the course of the simulation. Throughout the simulation, quercetin remained steadily bound at the active site of THBS1. The hydrogen bonds became increasingly stable over time, with a persistent hydrogen bond observed between quercetin and the ASN‐104 residue of THBS1. Additionally, van der Waals interactions were formed with residues such as GLN‐120, GLY‐185, and SER‐124. Electrostatic interactions played a dominant role in the binding, while van der Waals forces contributed secondarily, and hydrophobic interactions also provided complementary stabilization (Figure 6). During the simulation, minor fluctuations in van der Waals, hydrophobic, and electrostatic energies were observed, likely due to subtle conformational adjustments of the ligand within the binding pocket. Nevertheless, the consistently strong binding energy and high binding affinity indicated a robust interaction between quercetin and THBS1.

**FIGURE 7:**
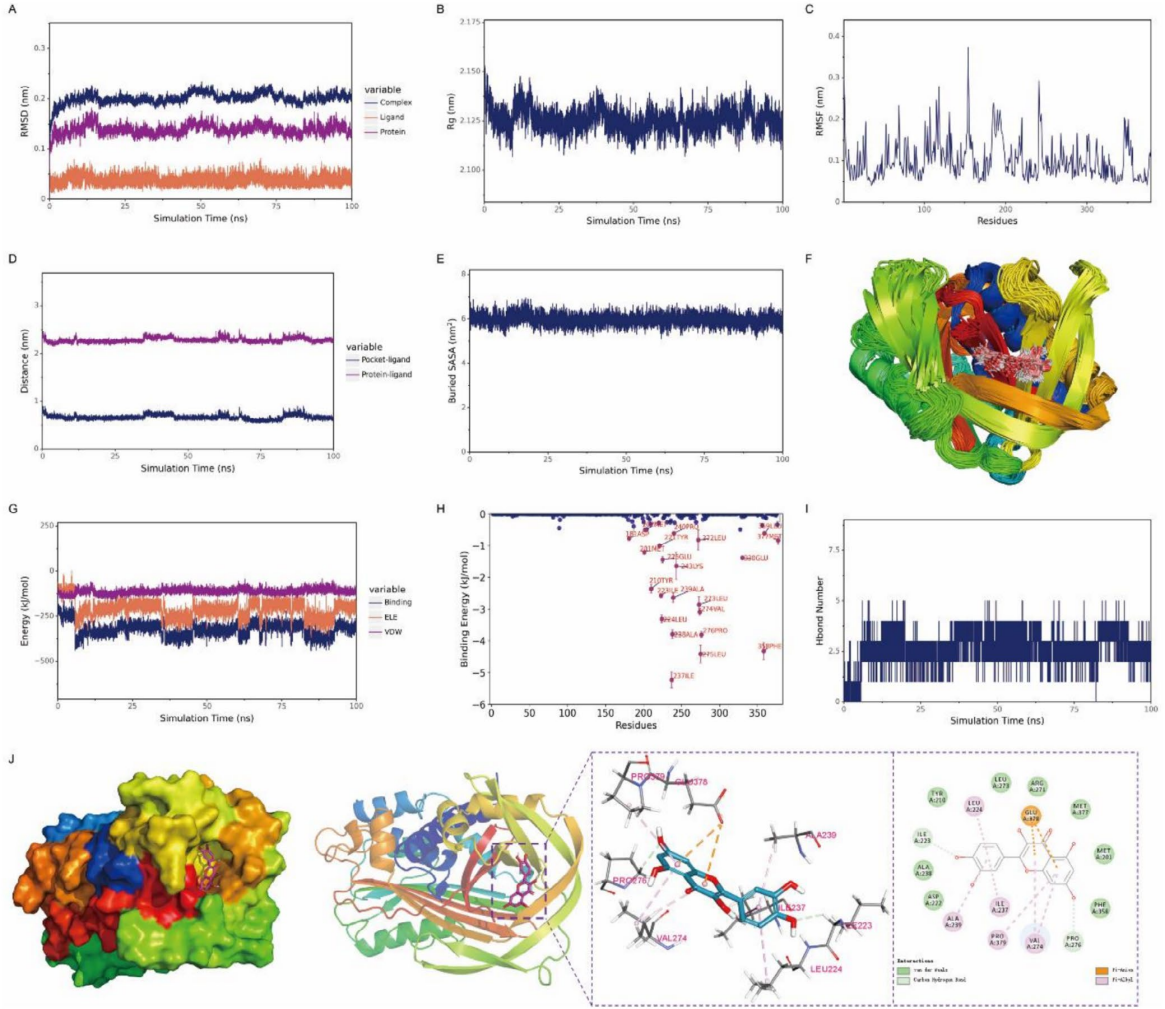
Molecular dynamics simulations demonstrate the stable binding of quercetin to the SERPINE1 protein. (A) RMSD of the complex, protein, and ligand; (B) Rg of the complex; (C) RMSF of protein residues. (D) Protein‐ligand binding site distance; (E) Buried SASA; (F) Superimposed conformational snapshots; (G) VDW and ELE energy components; (H) Binding energy contribution per residue; (I) Hydrogen bond number; (J) Molecular interaction diagram.

The binding of quercetin to the SERPINE1 protein was stable, with the complex structure becoming progressively stabilized during the simulation. Quercetin remained consistently bound to the active site of SERPINE1 throughout the simulation. The number of hydrogen bonds fluctuated between 0 and 4. Van der Waals interactions were observed between quercetin and residues ASP‐222, ARG‐271, and PHE‐358 of SERPINE1, while Pi‐Anion and Pi‐Alkyl hydrophobic interactions were formed with residues such as GLU‐378 and VAL‐274. The strong binding energy and high affinity indicated a robust interaction between the ligand and the protein. Energetic analysis revealed that van der Waals interactions played a dominant role in the binding, electrostatic interactions contributed secondarily, and hydrophobic interactions provided additional stabilization (Figure 7).

**FIGURE 8:**
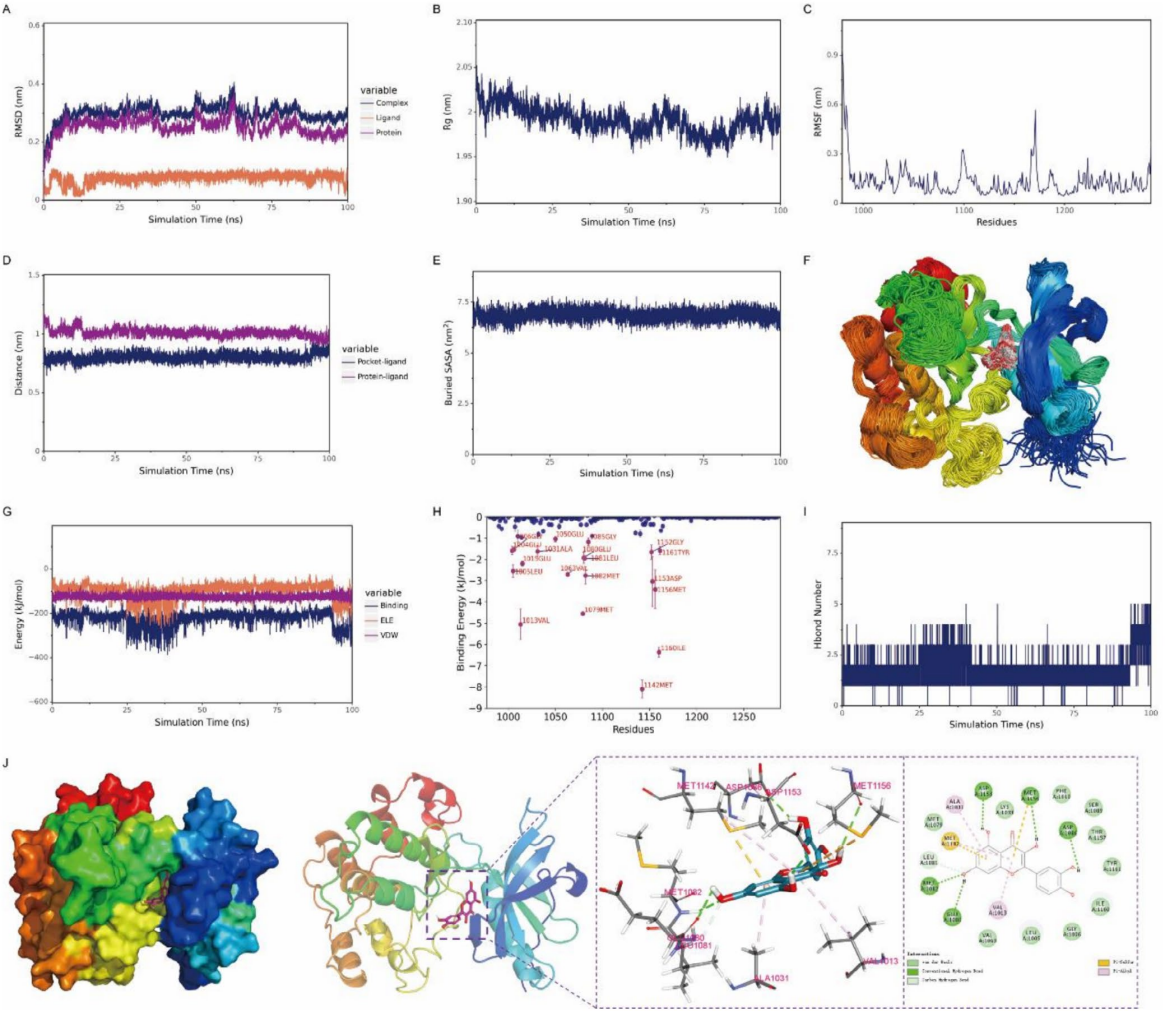
Molecular dynamics simulations demonstrate the stable binding of quercetin to the IGF1R protein. (A) RMSD of the complex, protein, and ligand; (B) Rg of the complex; (C) RMSF of protein residues. (D) Protein‐ligand binding site distance; (E) Buried SASA; (F) Superimposed conformational snapshots; (G) VDW and ELE energy components; (H) Binding energy contribution per residue; (I) Hydrogen bond number; (J) Molecular interaction diagram.

Quercetin exhibited stable binding to the IGF1R protein, maintaining consistent interaction within the active site throughout the simulation. The number of hydrogen bonds between quercetin and IGF1R fluctuated moderately, ranging from one to four. Key hydrogen bonds were formed with residues ASP‐1086, MET‐1082, and ASP‐1153. Additionally, van der Waals interactions were observed with MET‐1079, SER‐1086, and LEU‐1005, while hydrophobic interactions, including Pi‐Anion and Pi‐Alkyl types, were identified with residues such as MET‐1142 and MET‐1156. Energy decomposition analysis indicated that van der Waals interactions served as the primary driving force for binding, electrostatic interactions played a secondary role, and hydrophobic interactions provided complementary stabilization (Figure 8).

### 3.4 Experimental Verification of the Inhibitory Effect of Quercetin on Oral Cancer Cells and Transformed Cells

**FIGURE 9:**
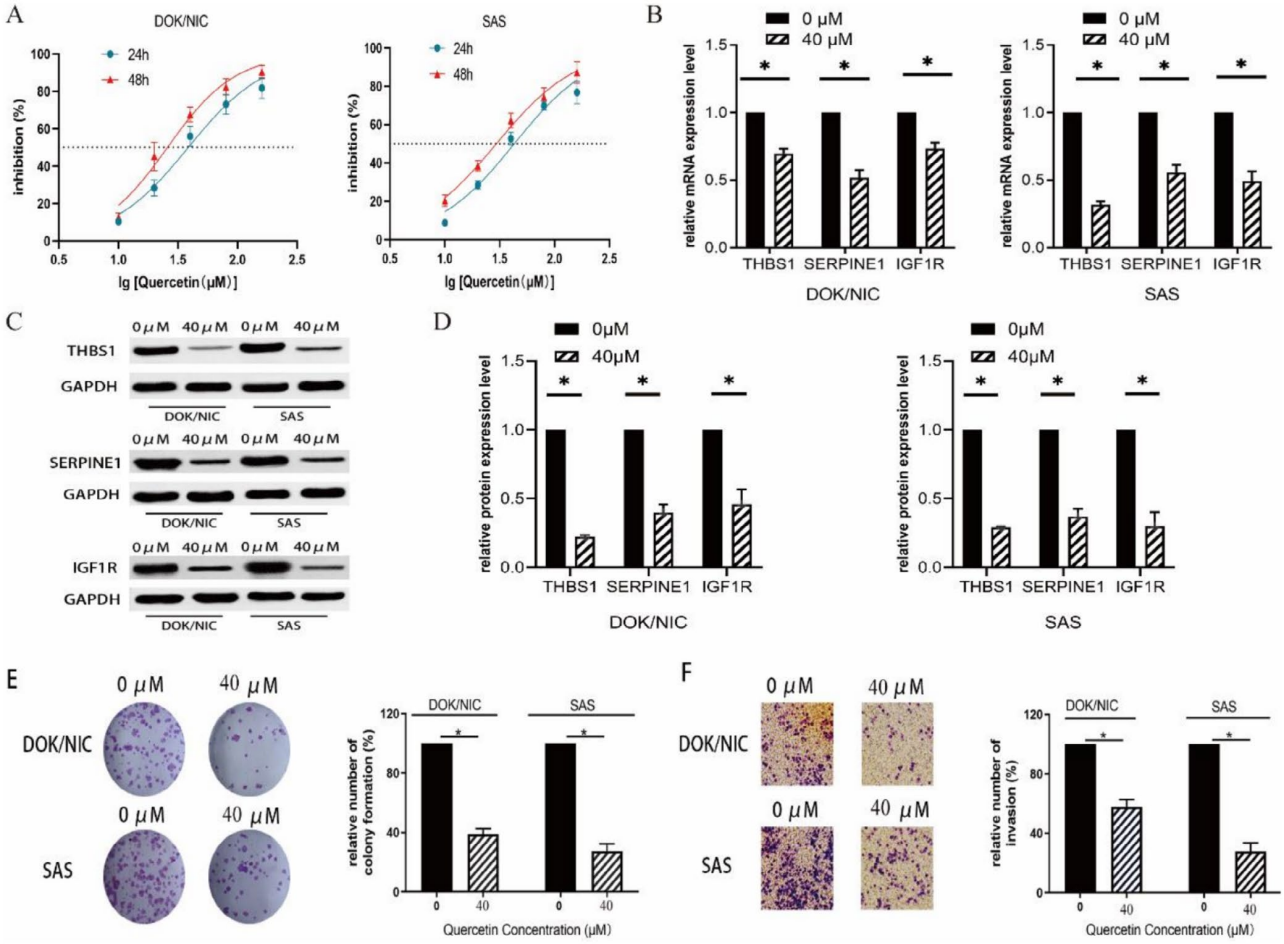
The experimental verification results of the inhibitory effect of quercetin on oral cancer cells and transformed cells. (A) The cell proliferation ability was evaluated using the CCK‐8 assay. Quercetin inhibited the proliferation of DOK/NIC and SAS cells in a concentration‐ and time‐dependent manner (*p* < 0.05). (B–D) The expression of mRNA and protein from key target genes in nicotine‐transformed oral epithelial cells and oral cancer cell lines was examined. After quercetin intervention, the mRNA (B) and protein (C,D) expression of THBS1, SERPINE1 and IGF1R were downregulated in DOK/NIC and SAS cells (*p* < 0.05). (E) The effect of quercetin on cell proliferation was evaluated using a clone‐formation assay. Quercetin treatment significantly reduced the clone formation of DOK/NIC and SAS cells (*p* < 0.05). (F) The effect of quercetin on cell invasion was evaluated using the Transwell assay. Compared to the control group, the invasive ability of cells significantly decreased after quercetin intervention (*p* < 0.05).

The cell viability of DOK/NIC and SAS cells was investigated using different concentrations of Quercetin for 24 and 48 h, respectively. The data showed that the administration of Quercetin suppressed the cell viabilities of both cell types in a time‐ and concentration‐dependent manner (Figure 9A), Quercetin exhibited IC50 values of 38.48 μM (24 h) and 25.75 μM (48 h) in DOK/NIC cells, and 42.82 μM (24 h) and 29.50 μM (48 h) in SAS cells. Taking into consideration the inhibitory efficacy across both cell lines and time points, a concentration of 40 μM (rounded to the nearest ten) was chosen for further investigations.

After subjecting the cells to a 40 μM concentration for 24 h, a significant inhibitory effect of Quercetin on both cell types was observed. Hence, we adopted this concentration and duration for subsequent experiments.

The mRNA and protein expression of the candidate genes (THBS1, SERPINE1, and IGF1R) was downregulated in both types of cells, respectively, after Quercetin treatment (Figure 9B–D, *p* < 0.05).

The results of the colony formation experiments demonstrated a significantly reduced capacity for cell clone formation in the Quercetin‐treated group compared to the control group, for both cell types (Figure 9E, *p* < 0.05). Additionally, the Quercetin‐treated group exhibited a significantly lower invasion ability than the control group (Figure 9F, *p* < 0.05).

## 4 Discussion

Oral medications need to overcome the absorption barrier before reaching the site of drug action. Therefore, OB is an important pharmacokinetic parameter. An OB value greater than 30 is usually considered a criterion for compound screening and is a necessary prerequisite for evaluating pharmacological activity. DL refers to the similarity between drugs and known drugs. The higher the DL value, the greater the potential of the compound as a drug. Generally, a DL value greater than 0.18 is considered a safety indicator for drug screening (Ling, Wang, et al. 2022; Liu et al. 2013). Our research results indicate that quercetin has an OB value of 46.43 and a DL value of 0.28, suggesting that quercetin can be effectively absorbed after oral administration and has great potential as a drug. Furthermore, molecular docking simulations revealed that quercetin binds to the core gene protein with a favorable binding energy, primarily through van der Waals forces, electrostatic interactions, and hydrophobic interactions. The formation of multiple hydrogen bonds further stabilized the complex, indicating a strong and stable interaction. Quercetin is also naturally present in various fruits and vegetables and can be employed as a dietary supplement, which can prevent or treat various chronic diseases including cancer (Deepika and Maurya 2022). This suggests that quercetin has the potential to serve as a dietary supplement for disease prevention and treatment.

Through network pharmacology studies, additional gene targets of quercetin for treating nicotine‐related oral carcinoma, such as THBS1, IGF1R, and SERPINE1, were identified. These core genes may have played an essential role in tumor development. For example, it was found that IGF1R acted as a cancer‐promoting factor in the tumor microenvironment, facilitating lung metastasis implantation and progression (Alfaro‐Arnedo et al. 2022). SERPINE1 overexpression promotes malignant progression, metastasis, and invasion of gastric adenocarcinoma and is associated with a poor prognosis of gastric cancer (Yang et al. 2019). In our previous study, SERPINE1 was screened as a key gene in nicotine‐induced oral cancer while being highly expressed in oral cancer and correlated with metastasis and prognosis of oral cancer (X. Guo et al. 2023). In the experimental validation, the proliferation and invasion activity of oral cancer cells treated with quercetin or cells transformed by nicotine were significantly inhibited. Thus, it can be inferred that quercetin might inhibit the nicotine‐induced malignant transformation of oral cells by targeting the key genes.

Quercetin has various biological activities, such as anti‐inflammatory, antioxidant, anti‐microbial, and anti‐tumor (Deepika and Maurya 2022). For antitumor activity, Quercetin can suppress tumor biological processes by regulating gene expression, such as inhibiting TLR‐4 to reduce cell migration and invasion ability and activating ERβ2 to induce tumor cell apoptosis (Maugeri et al. 2023), and modulating Let‐7, miR‐21, and miR‐155 to inhibit the occurrence and development of cancer (D. H. Kim et al. 2019). Furthermore, quercetin exerts its anticancer effects by regulating multiple pathways, such as PI3K/Akt/mTOR, Wnt/β‐linked protein, and MAPK/ERK1/2 pathways (Kim et al. 2020). In addition, quercetin exerts its antitumor properties by modulating mediators of cell death and autophagy in various cancer cells (Wang et al. 2023). Therefore, it can be seen that as a natural substance, quercetin has effects on multiple targets and pathways, and studying the effects of a single gene would be biased. GSVA is a non‐parametric and unsupervised analysis method that estimates the variation in gene set activity within a sample population. GSVA scores represent the comprehensive level of gene set expression (Hanzelmann et al. 2013). Therefore, we will analyze all the common genes that quercetin may act upon as a gene set. Based on the GSVA scores, we can comprehensively investigate the potential mechanisms of action of quercetin.

In the GSVA analysis, we found that the common genes are positively correlated with EMT and RTK, and negatively correlated with DNA damage. This result is consistent with the results of the single gene analysis. EMT refers to the process by which epithelial cells acquire mesenchymal characteristics, transforming less motile epithelial cancer cells into more migratory fibroblast‐like or mesenchymal phenotypes. EMT is associated with tumor development, invasion, metastasis, and treatment resistance (Su et al. 2019). For example, it has been found in studies that EMT is associated with the characteristics of tumor cancer stem cells and drug resistance (Pan et al. 2021), and inhibiting EMT can effectively control the occurrence and metastasis of breast cancer (Bai et al. 2021). Another study found that EMT is a crucial factor in the invasion and metastasis of oral squamous cell carcinoma (F. Wang et al. 2020). Our previous research also revealed that SERPINE1, which is highly expressed in oral cancer, is related to EMT, and suppresses the expression of SERPINE1 can significantly weaken the invasion ability of oral cancer (Guo et al. 2023). These research findings suggest that EMT plays an important role in regulating the enhancement of cell invasion and metastasis in oral cancer development. The RTK pathway is an oncogenic signaling pathway that can be a potential target for tumor immunotherapy (Yin et al. 2022). For instance, researchers have studied targeted RTK therapy for melanoma (Sabbah et al. 2021), as well as targeting the RTK‐PI3K‐mTOR pathway for glioblastoma treatments (Colardo et al. 2021). Additionally, we have also noted that certain common genes are associated with DNA damage repression, and higher GSVA scores indicate lower DNA damage pathway activity. This finding contradicts conventional wisdom, which holds that DNA damage leads to gene mutations, resulting in protein changes and ultimately cancer. However, studies have shown that cells respond to DNA damage by activating complex signaling networks that determine cell fate, promoting DNA repair and survival while also inducing cell death (Roos et al. 2016). We speculate that these genes may reduce genetic damage in cancer cells through such intricate pathways, thereby reducing cancer cell death. In summary, we can hypothesize that quercetin primarily acts on these hub genes to inhibit EMT and the RTK pathway, thus reversing the malignant phenotype of cancer cells.

Notably, these target genes may also be associated with the sensitivity of cancer cells to a range of anti‐cancer drugs. Among them, bleomycin and docetaxel have been studied and applied in clinical settings. Bleomycin is used as an electro chemotherapeutic drug for head and neck cancer treatment (Plaschke et al. 2017). Docetaxel is used as a radiosensitizer for locally advanced head and neck squamous cell carcinoma patients (Patil et al. 2023) or in neoadjuvant concurrent chemoradiotherapy for advanced oral cancer (Sato et al. 2019), which can improve patient prognosis. However, the increasing occurrence of chemoresistance has become a major challenge in oral cancer treatment (Sha et al. 2021). Understanding and overcoming this problem has become a primary goal for many researchers. For example, studies have found that increased HBEGF expression may induce EMT and enhance metastasis, which could be a mechanism of cetuximab resistance in head and neck cancer (Hatakeyama et al. 2010). Additionally, CDH1 involvement in the EMT process, impacting the proliferation, invasion, and sensitivity to cisplatin in oral cancer cells (Su et al. 2019). It has been reported that quercetin has the ability to reverse chemoresistance in certain cancers. For instance, it reverses docetaxel resistance in prostate cancer through the androgen receptor and PI3K/Akt signaling pathway (Lu et al. 2020), and reverses multi‐drug resistance in hepatocellular carcinoma cells through the FZD7/β‐catenin pathway (Chen et al. 2018). In summary, we propose that quercetin may reverse drug resistance in oral cancer by modulating the expression of hub genes, particularly through pathways involved in EMT. However, the specific mechanisms of action are not yet clear and need further investigation in future studies.

We have observed a potential correlation between the expression of the common genes and immune cell infiltration in the tumor microenvironment. These immune cells encompass various types, such as macrophages, neutrophils, NKT cells, dendritic cells, regulatory T cells, effector T cells, B cells, CD8+ T cells, γδ T cells, and others. The presence of immune cells within the TME is pivotal in tumor development (Lei et al. 2020). Some of these immune cells exhibit anti‐tumor effects. For instance, CD8+ T cells are a major effector cell type in anti‐cancer immunity (He et al. 2019), and γδ T cells exhibit strong cytotoxicity and pro‐inflammatory activity, enabling them to eliminate a wide range of tumor cells. Infiltration of γδ T cells in tumors is also considered a positive prognostic marker (Imbert and Olive 2020). Research has shown that suppression of CD8+ T cell exhaustion can hinder tumor development (Kumar et al. 2021), and clinical application of allogeneic γδ T cell immunotherapy has demonstrated prolonged survival in patients with advanced lung or liver cancer (Xu et al. 2021). NKT cells exhibit anti‐tumor activity by regulating the frequency of M1 macrophages and effector Th1 cells in secondary lymphoid tissues and the tumor microenvironment, thereby suppressing solid tumor growth (Paul et al. 2019). DCs play a dual role: they are essential for activating effective anti‐tumor T‐cell responses but can also be influenced by tumor‐mediated factors to promote immune tolerance and cancer progression (Heras‐Murillo et al. 2024). In summary, this study found that the key genes were negatively correlated with the infiltration of anti‐tumor immune cells such as γδ T cells and CD8+ T cells, but positively correlated with NKT/DC cells. Given the complexity of tumor immune infiltration and cellular interactions, we propose that quercetin's antitumor effect is not reliant on a single immune cell type. Instead, it is likely mediated through the regulation of these key genes, thereby comprehensively reshaping the overall immune microenvironment. Although the precise mechanism requires further elucidation, the net effect of quercetin is anti‐tumorigenic. Thus, the mechanism of quercetin's effect on immune cell infiltration remains to be explored in our future work.

This study may have several limitations. First, multiple databases were used for acquiring the target genes of quercetin. Due to algorithmic differences, some target genes may have been inevitably ignored during the screening process; meanwhile, using head and neck tumors to represent oral carcinoma in some bioinformatics analyses may lead to certain discrepancies, potentially leading to any bias in the results. Second, the specific mechanisms by which quercetin affects immune‐infiltrating cells require further experimental verification. Third, only in vitro experiments were performed for experimental validation, the concentration and effect of quercetin achievable in vivo have not been verified. Therefore, future investigations are needed to provide insights into the role and mechanisms of quercetin intervention in developing nicotine‐related oral carcinoma.

## 5 Conclusion

Despite these limitations, the current study suggests that quercetin may be a potential adjuvant therapeutic compound for oral cancer by modulating the expression of target genes, while improving the infiltration of immune cells in oral cancer and increasing the sensitivity of cancer cells to chemotherapeutic agents. In addition, quercetin may also inhibit the carcinogenic process of nicotine by interfering with the expression of these target genes, suppressing the occurrence of oral cancer at the pre‐cancerous stage, which may be of great significance for its cancer prevention.

## Author Contributions

**Xiaopeng Guo:** conceptualization (equal), data curation (equal), formal analysis (lead), validation (equal), visualization (equal), writing – original draft (lead), writing – review and editing (equal). **Zhen Sun:** data curation (equal), formal analysis (equal), investigation (equal), visualization (equal), writing – review and editing (equal). **Huarong Chen:** formal analysis (equal), validation (equal), visualization (supporting). **Changya Li:** formal analysis (equal), validation (supporting), visualization (supporting). **Aoshuang Chang:** formal analysis (equal), investigation (equal), validation (supporting). **Houyu Zhao:** conceptualization (equal), funding acquisition (equal), project administration (equal), supervision (equal). **Junjun Ling:** conceptualization (equal), methodology (equal), validation (lead), writing – review and editing (equal). **Xianlu Zhuo:** conceptualization (lead), funding acquisition (lead), project administration (lead), supervision (lead), writing – review and editing (equal).

## Funding

This study was supported by Guizhou Provincial Basic Research Program (Natural Science) (No. 2022‐044, 2023‐045 and 2023‐327) and Guizhou Provincial Graduate Research Fund Project (No. 2024YJSKYJJ272 and 2024YJSKYJJ273).

## Ethics Statement

The authors have nothing to report.

## Conflicts of Interest

The authors declare no conflicts of interest.

## Data Availability

The data of this research result can be provided by the corresponding author upon reasonable request.
